# Time-related meal patterns and breakfast quality in a sample of Iranian adults

**DOI:** 10.1186/s40795-022-00666-w

**Published:** 2023-01-16

**Authors:** Azadeh Lesani, Bahareh Barkhidarian, Mehrzad Jafarzadeh, Zahra Akbarzade, Kurosh Djafarian, Sakineh Shab-Bidar

**Affiliations:** 1 Department of Community Nutrition, School of Nutritional Sciences and Dietetics, Tehran University of Medical Sciences, Tehran, Iran; 2 Endocrine Research Center, Institute of Endocrinology and Metabolism, Iran University of Medical Science, Tehran, Iran; 3 Department of Clinical Nutrition, School of Nutritional Sciences and Dietetics, Tehran University of Medical Sciences, Tehran, Iran

**Keywords:** Meal timing, Breakfast quality, Eating window, Fasting window, Chronotype

## Abstract

**Background:**

Some evidence shows that meal timing is associated with diet quality. We aimed to investigate the relationship between meal timing and breakfast quality in a sample of Iranian adults.

**Methods:**

This cross-sectional study was conducted on 850 men and women living in Tehran, Iran. Dietary data was recorded by three non-consecutive 24-h dietary recalls. The Breakfast Quality Index (BQI) was calculated. Time-related meal patterns included the interval between the first and last meal (eating and fasting window), frequency of meals, length of sleep, and time of first and last eating occasion. The multiple linear regression analysis was used to identify the relationships between time-related meal patterns and BQI.

**Results:**

The mean (95% CI) of BQI was 4.52 (4.45–4.65) and the maximum was 8. Bread, cheese, vegetables, fruits, energy, and carbohydrate intake showed positive associations with BQI scores. Longer fasting time showed a positive association with fruits (β (CI 95%)) (0.11 (0.0003–0.005), and vegetable consumption (0.12 (0.009–0.07)) and BQI score (0.39 (0.001–0.06)). Time of first eating occasions indicated a negative association with protein and fat intake and BQI score. Time of last eating occasions showed a positive association with vegetables consumption and BQI score. The longer length of sleep was associated with a higher BQI score. The frequency of meals was significantly and positively related to energy and macronutrients intake and BQI.

**Conclusion:**

Earlier first and last meal, longer sleep length, longer fasting window, and a greater meal frequency were associated with a better breakfast quality.

## Background

Recent nutrition research has focused on the link between nutrition and the biological clock or circadian rhythm [1, 2]. These studies have indicated the timing of food intake has some physiological and nutritional consequences. Feeding time could have some physiological benefits, like a protective effect against obesity and metabolic outcomes in a single mouse model by a high-fat diet [3]. It has also been shown that meal timing [4, 5], sleep timing [6, 7], eating window, the interval between the first and last mealtime [8], fasting window, the interval between the last and first meal as time-restricted feeding [9] and irregularity in intake of energy at meals especially breakfast [10] have an important role in weight control. Breakfast is usually known as the first meal of the day and an important component of a healthy eating pattern [11]. Previous studies have indicated that regular breakfast eaters had better diet quality [12], lower obesity risk [12, 13], and better well-being [14]. Breakfast consumption could improve energy balance, and lower metabolic imbalance [13] because of the effect of meal timing on the circadian pattern [15]. Some previous studies focused on the association of breakfast quality with obesity [12] and metabolic disorders [16]. Meal timing could also affect health outcomes. It was reported that one hour increase in the timing of the first and last meals is related to higher inflammation, insulin concentration, and hemoglobin A1c [17]. According to a report from Djuric et al., early breakfast and dinner eaters had better diet quality in a Serbian population. In addition, intake of more calories before 16:00 is related to higher intake of fruits and vegetable and diet quality scores [18]. Similar results were indicated by Lima and et al., in which early breakfast and dinner consumption and early midpoint eating was related to better scores for specific Brazilian healthy eating index among females [19]. Late dinner or bedtime snack consumption may be related to the skipping breakfast [20]. In addition, previuse studies showed skipping breakfast is associated with a lower diet quality [21, 22]. A recent study reported that the time of breakfast intake could be a good estimator of chronotype. Chronotype refers to a person’s preferences with regard to the timing of activities and sleeps [23]. People with evening chronotype may have a later breakfast time [24], skipping breakfast [25], late eating habits [26], lower energy and macronutrient intake in the morning [27], and lower intake of daily whole grain, fruits and higher refined grains [28], and process food [29]. In contrast, the morning chronotype is associated with a higher adherence to healthy dietary patterns [30] and lifestyle [31]. The recent literature review [32] and some previous studies indicated that time-related meal patterns may change the quality of diet and energy balance [5, 33]. According to our knowledge, this study is the first study that aimed to investigate the association of meal timing and chronotype with breakfast quality in the Iranian community.

## Methods

### Study design

A cross-sectional study was conducted among apparently healthy (those who do not have specific diseases according to the self-report and people who do not have physical problems in appearance) males and females from Iran who attended the healthcare center of Tehran (February 2019 to August 2019). A sample size of 493 was calculated based on the formula *n* = ((z_α_ + z_β_)/(0.5 × In [(1 + r)/(1-r)]))^2^ + 3 [34], according to correlation between eating frequency and energy intake *r* = 0.2 [35], at α level of 0.05 and 1-β 99%. Considering effect design 1.7, the final sample size of 850 participants was estimated for this study. Participants were recruited using two-stage cluster sampling from 5 geographic areas of Tehran within 25 healthcare centers. A convenient sampling method was used to select the study participants from each health centre, using the proportion-to-size approach. The inclusion criteria were being 20–59 years old and having a body mass index (BMI) of 18.5–39.9 kg/m^2^. The exclusion criteria were being pregnant or lactating, with under- and over-reporting of total energy intake, and individuals who had been diagnosed with acute disease.

### Ethical approval

Sample collection was fascinated by coordinating with the health care centers of Tehran. The study was ethically approved by the Ethics Committee of Tehran University of Medical Sciences (Ethics Number: IR.TUMS.MEDICINE.REC.1399.295). The methods were conducted in accordance with the relevant Declaration of Helsinki guidelines and regulations. The purpose of the study was explained to the participants, and all participants were given written informed consent precede to enter the study.

### Dietary intake assessment

Dietary data were obtained using 3-day repeated 24-h dietary recalls (24-hDRs). We collected all recalls by trained interviewers who encouraged the participants to describe all consumed foods for the previous day, from the first to the last meal. The first 24-hDRs was recorded in the first visit to the healthcare centre. The following 24-hDRs were collected via telephone on a random day. Meals and snacks were known as occasions where large amounts of energy contain food (at least 50 kcal) were consumed and were standardized based on time (at least 15 min intervals by prior and posterior eating occasion) [36, 37], also they were standardized to contain no more than one breakfast, lunch, and dinner, but allowing for multiple snacks. Breakfast was defined as the largest meal between 5:00–11:00 [38]. Daily intakes of all food items were derived from 24-hDRs and converted into grams by using household measures [39]. Average of foods over 3-day in breakfast used to derive breakfast intake. Dietary intake was adjusted for energy intake by the residual method [40].

### Time-related eating pattern

Time-related eating patterns were assessed through the frequency of meals or eating occasions (EO), time of the first and last meal, eating window, and nightly fasting window. The number of meals was defined by the number of caloric events ≥ 50 kcal/day with time intervals between meals of > 15 min, which was reported in the 24-hDRs. The time of the first and last meal was reported at the time of the 24-hDRs. The eating window was defined by the length between the first and last caloric event in the 24-hDRs [8]. The nightly fasting window was defined by calculating the hours between the first and last eating occasion for each day and subtracting this time from 24 h. These variables were calculated from the average of the 24-hDRs. The average 3-day time of interval time of going to bed and waking up is known as sleep length which was measured. Time-related pattern data was shown in Table 1.

**Table 1:** Sociodemographic, anthropometric and chronobiological data of participants by sex and age groups

	**Total**	**Women**		**Men**	
		Age 20–40 y	Age 40–60 y	Age 20–40 y	Age 40–60 y
**Number**	850	328	375	77	70
**Age**	42.15 ± 10.6	33.22 ± 5.53	50.76 ± 6.42	32.16 ± 6.12	48.74 ± 5.79
**Smoking status**					
**Current smoker**	28 (3.2%)	7 (1.8%)	5 (1.3%)	10 (13%)	7 (10%)
**Non-smoker**	822 (96.8%)	371 (97.2%)	325 (98.7%)	67 (87%)	63 (90%)
**Educational level**					
**Diploma and under diploma**	553 (65.05%)	176 (53.8%)	311 (82.7%)	31(40.3%)	35 (50%)
**Educated**	297 (34.95%)	151 (46.2%)	65 (17.3%)	46 (59.7%)	35 (50%)
**Occupation level**					
**Employed**	308 (36.4%)	115 (35.2%)	79 (21%)	59 (76.6%)	55 (78.6%)
**Un employed**	494 (58.1%)	208 (63.6)	259 (68.9%)	18 (23.4%)	9 (12.9%)
**Retired**	47 (5.5%)	4 (1.2%)	37 (9.8)	0	6 (8.9)
**Body Mass Index (BMI)**	27.20 ± 4.46	26.13 ± 4.26	28.13 ± 4.25	26.03 ± 5.11	28.49 ± 4.36
**Waist circumference**	87.82 ± 11.42	84.69 ± 11.53	89.72 ± 10.08	87.44 ± 12.62	92.62 ± 12.44
**Sleep duration (h:min)**	8:36 ± 1:34	8:36 ± 1:33	8:34 ± 1:31	8:52 ± 1:47	8:29 ± 1:41
**Nightly fasting window**^a^ **(h:min)**	9:32 ± 1:18	9:29 ± 1:13	9:33 ± 1:19	9:15 ± 1:16	9:57 ± 1:32
**Eating window**^b^ **(h:min)**	13:46 ± 1:35	13:43 ± 1:37	13:48 ± 1:34	13:44 ± 1:28	13:49 ± 1:42
**First meal time (h:min)**	7:35 ± 1:04	7:36 ± 1:04	7:33 ± 1:06	7:35 ± 1:02	7:39 ± 1:03
**Last meal time (h:min)**	21:21 ± 1:06	21:19 ± 1:07	21:21 ± 1:06	21:20 ± 1:02	21:26 ± 1:11
**Number of meals**	6.32 ± 0.89	6.36 ± 0.86	6.33 ± 0.91	6.31 ± 0.95	6.15 ± 0.79
**Chronotype** ^c^					
**Morning-type**	435 (50.2%)	163 (49.8%)	193 (51.3%)	41(53.2%)	38 (54.3)
**Indifferent- type**	340 (41%)	134 (41%)	152 (40.4%)	27 (35.1%)	27 (38.6%)
**Evening-type**	75 (8.8%)	30 (9.2%)	31 (8.3%)	9 (11.7%)	5 (7.1%)
**Participants skipped breakfast** ^d^	78 (9.5%)	30 (9.8%)	29 (8%)	9 (11.7%)	10 (14.5%)
**Misreporting (EI:BMR)**^e^					
**EI:BMR < 1.35**	23 (2.6%)	7 (2.1%)	7 (1.8%)	5 (6.1%)	4 (5.5%)
**1.35 ≤ EI:BMR > 2.40**	850 (97.1%)	325 (97.6%)	375 (98.1%)	77 (93.9%)	70 (94.5%)
**EI:BMR ≥ 2.40**	2 (0.3%)	1 (0.3%)	1 (0.2%)	0	0

Values are presented as number (percentage) or mean SD (standard deviation) in total population and according gender and age categories

^a^ Nightly fasting window was determined by hours between the first and last eating episode and the mean of three-day fasting window was measured

^b^ Eating window was determined in the length between firs and the last eating occasion for a day then the mean of three-day report was calculated

^c^ Chronotype was derived from morning evening questionnaire (MEQ) score in range 16–86, Morning-type (coefficient: 59–86), Indifferent-type (42–58) and Evening-type (16–41) [41]

^d^ participants skipped breakfast at least one day of three days

^e^ Misreporting was defined as ratio of Energy Intake (EI): Basal Metabolism Rate (BMR), EI:BMR < 1.35 was defined as under-reporting and EI:BMR ≥ 2.40 as over-reporting

### Chronotype

The Morning Evening Questionnaire(MEQ), a self-assessment questionnaire, was developed primarily for screening individual sleep-related experiments to the circadian rhythm or sleep rhythm pattern [42]. MEQ consists of 19 items on sleep habits and fatigue. scoring was according to the original questionnaire by Ostberg [42]. Eleven questions allowed for choice, which scored from 1 to 4, two questions scored 0,2,4 and 6. One question scored 0,2,3 and 5. Five questions scored from 1 to 5. The sum of all scores converted into five-point MEQ scores 1) definitely morning type (score 70–86), 2) moderately morning (score 59–69), 3)intermediate (neither) type (score 42–58), 4) moderately evening type (score 31–41), 5) definitely evening type (score 16–30). In the current study, we decreased categories from 5 to 3, morning type score 59–86, intermediate type score 42–58, and evening type score 16–41 [43]. Lower values indicated greater eveningness, and higher values showed greater morningness. A validated Persian version of MEQ was used [44]. Chronotype characteristic was reported in Table 1.

### Breakfast quality index

The breakfast quality index (BQI) has been developed to be a tool to assess the nutritional quality of breakfast at individual and population levels [45, 46]. The BQI involves ten components, food groups, energy and nutrients of public health concern, with scores of (0 or 1) for each component and a maximum possible score of 10. The three food group components of BQI include cereals, fruits or vegetables, and dairy products. No points were removed for unhealthy foods consumed at breakfast, such as processed meats and industrialized juices. Mixed foods were counted in multiple categories based on their components. The scoring system for the food group components was qualitative; for example, we did not consider the amounts consumed and only considered whether the food group was reported as being consumed or not on dietary records [45]. So, if an individual reported consuming the food group in only one or both dietary records, the participant scored one point. While a participant did not report the consumption of the food group, the individual scored zero on that group. Also, the fourth component according to the combined consumption of cereals, dairy products and fruit or vegetables at breakfast on at least one day was included. Individuals who consumed only non-caloric beverages at breakfast on both days (like coffee, tea, and diet soda) scored zero points in the BQI. Unlike the scoring system of the food group components, the scores for energy and nutrient components were based on quantitative criteria. The BQI energy and nutrient components are breakfast energy intake (15–25% of total daily energy intake) [46] and free sugar intake at breakfast (< 10% total daily energy divided by the number of daily EO of the participants, calcium intake (20% of the recommended dietary allowance (RDA) according to participants’ life stage group) [47]. Fiber intake was extracted from nutritionist 4 (N4) software (> 25 gr divided by the number of daily EO of the individual and sodium intake (< 2000 mg divided by the number of daily EO of the individuals, as proposed by O'Nei, et al. [46]. The BQI scores were divided to the three groups: low (0–3 points), medium (4–6 points), and high (≥ 7 points).

### Demographic and anthropometric data

Data were collected by trained interviewers. Sociodemographic characteristics included age, gender, smoking status (current smoker and non-smoker), educational level (diploma and under diploma and educated), and occupation(employed, unemployed and retired). Body weight was measured when wearing light clothes to the nearest 0.1 kg by a digital Seca scale with a measurement accuracy 100 g [48]. Height was measured in a standing situation, shoulders, and barefoot touching the wall to the nearest 0.5 cm. Body mass index (BMI) was calculated by dividing weight in (kg) to height in (m^2^).

### Statistical analysis

Data analysis was done by Statistical Package for Social Sciences (SPSS) version (version 22:0, SPSS Inc., Chicago, IL). The Kolmogorov–Smirnov test was used to examine the normal distribution of variables. The demographic characteristics of participants were compared by using χ2 for categorical variables and analysis of variance (ANOVA) for continuous variables across BQI categories. T-test was used to compare gender differences in energy and nutrient intake among age groups. Multiple linear regression analysis was used by controlling confounders (age, gender, physical activity, educational level, occupation, smoking status, energy intake, supplement intake, and BMI) to find the association between food groups, macronutrients, and energy intake and (BQI) score and time-related patterns (fasting window, eating window, first time of eating occasion, last time of eating occasion, sleep length, frequency of meals). Misreporting was measured by the ratio of energy intake (EI) to basal metabolism rate (BMR) based on Harris benedict formula. EI:BMR < 1.35 as underreporting, EI:BMR ≥ 2.40 as overreporting were defined [49, 50].

## Results

This cross-sectional study was conducted on 877 Iranian adults of both genders. 27 participants were excluded due to misreporting (*n* = 25) and not having breakfast (*n* = 2) at any 24hDRs. Finally, all analyses were conducted on 850 participants (147 males (17.29%) and 703 females (82.71%). The mean (SD) age was 42.15 ± 10.6 (range of 20–60 years old) and the mean (SD) BMI was 27.2 ± 4.51 kg/M^2^. Out of 850 participants, 799 individuals (94%) consumed breakfast in all 24-hDR and 51 individuals (6%) skipped breakfast at least one day out of the 3-day dietary reports. Mean (SD) of breakfast time was 8:02 ± 0:44 (range 6:10 – 10:45) (hours:minutes). The mean (SD) of time-related pattern was 7:10 ± 1:26 for the length of nightly sleep, 7:34 ± 1:05 for the time of first eating occasion, 21:36 ± 1:04 for the time of last eating occasion, 10:41 ± 1:20 for fasting window, and 13:07 ± 1:20 for eating window. The mean meal frequency was 6.31 ± 0.89 Table 1 shows demographic characteristics and time-related pattern data in the population.

Table 2 indicates the percentage of Iranian adults who scored for each BQI component in the total population and across categorized BQI. Cereal and derivatives were the most prevalent food component consumed by 796 participants (93.65%), which scored positively, followed by dairy products by 757 participants (89.11%), and then 45.18%, 385 of participants for consumption of fruit or vegetables. 39.59% of participants (336 individuals) consumed cereal, fruit or vegetables and dairy products together at breakfast. BQI scores indicated that the majority of the participants had a score of 4–7 points (529 participants, 62.2%) as a medium, while 27.2%, 231 individuals had scores ranging from 0 to 3 as low and 90 participants approximately (10.6%) had scored ranging from 7 to 10 as high breakfast quality.

**Table 2:** Description of Breakfast Quality Index (BQI) component and distribution of score point for each component across BQI categories in Iranian adults

BQI components	**Total population**	**BQI categories**			***P* value**
		**Low**	**Medium**	**High**	
		**(0-3points)**	**(4–6 points)**	**(7–10 points)**	
	*n* = 850	***n* = **231	***n* = **529	***n* = **90	
	% (95%CI)	**% (95%CI)**	**% (95%CI)**	**% (95%CI)**	
**1. Cereals and derivatives consumption** ^a^	93.65 (92.13—95.43)	84.32 (79.01—89.43)	96.33 (94.10—97.85)	96.05 (92.54—99.61)	< 0.001
**2. Fruit or Vegetables consumption** ^b^	45.18 (42.32—48.56)	6.83 (3.75—10.74)	53.3 (48.17—55.48)	88.01 (84.33—91.09)	< 0.001
**3. Dairy products consumption** ^c^	89.11 (87.50—91.51)	72.30 (66.34—79.13)	94.35 (93.19—95.45)	97.23 (96.43—99.01)	< 0.001
**4. Cereal, Fruit or Vegetables and Dairy products consumption in the same meal**	39.59 (36.37—43.56)	0	27.34 (22.94—32.77)	96.43 (91.32—99.75)	< 0.001
**5. Compliance with energy intake recommendations (15–25% of total daily energy)** ^d^	28.61 (25.64—32.86)	10 (6.03 -14.44)	32.38 (25.71- 36.22)	54.55 (51.65—57.77)	< 0.001
**6. Free sugar content (< 10% total daily energy divided by the number of daily eating occasion of the individuals**	49.34 (46.58—53.02)	29.02 (23.37- 35.80)	54.20 (49.91- 58.01)	74.55 (70.39 -78.41)	< 0.001
**7.Calcium content (20% of daily value)** ^e^	42.50 (39.21- 45.80)	22.80 (16.81- 28.53)	42.38 (41.63—49–81)	43.40 (42.30—53.90)	< 0.001
**8. Saturated fat content (< 10% total energy intake mg divided by the number of daily eating occasion of the individuals**	39.41 (36.21—42.61)	19.1 (13.50—24.02)	44.43 (39.05—48.11)	44.33 (39.21—49.04)	< 0.001
**9. Total fibre content (> 25 gr divided by the number of daily eating occasion of the individual)**	26.01 (23.44—29.48)	11.43 (7.19—13.66)	27.11(22.72—32.54)	42.33 (36.63—48.90)	< 0.001
**10. Sodium content (< 2000 mg divided by the number of daily eating occasion of the individuals**	42.54 (38.90—45.56)	25.02 (19.10—33.85)	42.22 (37.45—47.71)	61.46 (60.43—69.43)	< 0.001

Data are presented in frequency (%) confidence interval 95% (CI 95%), Differences across BQI categories were assessed by using Pearson's Chi-square test

^a^ Includes breads, oats, oat flour, oatmeal, quinoa, wheat, granola, corn flakes, cereal flakes, cookies and crackers, bakery products, cakes

^b^ Includes all fresh fruits and vegetables, consumed alone or in combination (e.g., fruit salad, fruit smoothie), excluding juices

^c^ Includes milk and milk drinks, coffee with milk, yogurt, cheese, fruit smoothie

^d^ According to WHO Recommendations for Prevention of Chronic Diseases 2003 [51]

^e^ Daily value based on Recommended Dietary Allowances (RDAs) for life stage groups (DRI, 2010)

Table 3 shows the distribution of demographic and anthropometric characteristics by categories of BQI. The mean of BQI scores was 4.58 for the overall population, also by sex specified was 4.86 in males and 4.68 in females. BQI score showed a significant difference among adults aged 20–40 and 40–60, so younger adults had higher BQI scores. BQI scores did not show any significant association by sex, smoking status, educational level, BMI, supplement intake, and chronotype.

**Table 3:** Mean BQI score and distribution of participants in BQI categories according to demographic, socioeconomic and anthropometric characteristic in Iranian adults

	**BQI score** **Mean (95%CI)**	***P* value**	**Total population** **n (%)**	**% distribution of participants according BQI categories**			***P* value **^b^
				Low	Medium	High	
				(0-3points)	(4–6 points)	(7–10 points)	
			*n* = 850	*n* = 231	*n* = 529	*n* = 90	
			n (%)	%	%	%	
**Gender**		0.199					0.583
Males	4.86 (4.59–5.10)		147 (17.3)	25.4	64.2	10.1	
Females	4.68 ( 4.56–4.79)		703 (80.7)	26.8	61.3	11.9	
**Age**		0.161					0.047*
20–40 y	4.79 (4.64–4.94)		404 (47.5)	25.8	61.3	12.9	
40–60 y	4.64 (4.49–4.78)		446 (52.5)	28.6	62.4	9.1	
**Smoking status**		0.301				5	0.571
Non-smoker	4.71 (4.60–4.82)		802 (94.4)	27.4	62.1	10.5	
Ex-smoker	4.30 (3.61–4.98)		20 (2.4)	25	70		
Current smoker	5 (4.47–5.52)		28 (3.2)	28.4	60.9	10.7	
**Educational level**		0.51					
Diploma and under diploma	4.53 (4.41–4.66)		553 (65.55)	76.1	64.6	63.7	0.725
Eeducated	4.56 (4.38–4.74)		297 (34.95)	32.9	35.4	36.3	
**Body Mass Index (BMI)**		0.45					0.673
Underweight	4.09 (3.11–5.06)		11 (1.3)	27.1	72.9	0	
Normal weight	4.78 (4.60–4.96)		283 (33.3)	25.6	62.2	12.2	
Overweight	4.70 (4.53–4.85)		341 (40.1)	27.9	63.5	9.6	
Obese	4.67 (4.45- 4.89)		215 (25.3)	29.1	59.8	11.1	
**Supplement intake**		0.06					0.274
Yes	4.76 (4.64- 4.88)		201 (23.6)	28.1	61.2	10.7	
No	4.53 (4.32- 4.75)		649 (76.4)	27.3	63.2	9.5	
**Chronotype**		0.08					0.616
Morning type	4.63 (4. 44- 4. 88)		435 (50.2%)	25.5	44.4	30.1	
Indifferent type	4.47 (4.54- 4.69)		340 (41%)	28.8	44.1	27.1	
Evening type	4.34 (4.17- 4.55)		75 (8.8%)	29.3	48	22.7	

^a^ Mean and confidence interval 95% (CI 95%) are presented. T-test (comparing two groups) and One Way ANOVA (comparing three or more groups) analysis were used

^b^ Differences of BQI categories were assessed by using Pearson's Chi-square test

^*^ Significant *P* value > 0.05

Table 4 shows the mean intake of energy and nutrients considered in the BQI by sex and age groups. The intake of energy at breakfast (*P* = 0.029) and the propotion of breakfast energy in daily energy intake (*P* = 0.031) was different between male and females. Nutrient intake did not indicate any significant difference by age and sex groups.

**Table 4:** Breakfast nutrient intake (mean and 95% confidence interval (CI)) by age group and sex and relationship with percentage of total daily energy intake. Iranian adults 20–60 years old

Breakfast nutrient	Women		Men		Mean intake / B	Mean intake /D
	**20–40 years** ***N* = 351**	**40–60 years** ***N* = 352**	**20–40 years** ***N* = 75**	**40–60 years** ***N* = 72**		
	**Mean (95%CI)**	**Mean (95%CI)**	**Mean (95%CI)**	**Mean (95%CI)**	**Mean (95%CI)**	**Mean (95%CI)**
Energy*	432.04 (415.6 – 448.49)	432.7 (421.55 – 498.12)	471.41 (437.04 – 505.78)	460.8 (419.56 – 500.12)	416.1 (411.6—419.4)	1686.4 (1659.80 -1813.3)
Breakfast energy v. daily energy* (%)	24.53 (23.79 – 25.19)	24.82 (24.03 – 25.61)	26.26 (24.32—27.41)	26.27 (24.25 -28.21)	-	-
**Carbohydrate (g)**	69.73 (66.41—73.9)	69.4 (66.53 – 72.08)	76.08 (70.26—81.90)	75.67 (68.24 – 83.09)	66.48 (64.80—69.35)	248.22 (241.35—257.09)
**Lipids(g)**	13.09 (12.28 – 13.9)	14.09 (12.92 – 15.26)	14.05 (12.81 – 15.67)	12.61 (11.24 -13.99)	13.03 (12.31- 13.86)	56.48 (55.26—59.68)
**Proteins(g)**	12.95 (12.17- 13.73)	13.02 (12.51 – 13.53)	13.21 (12.16 -14.26)	12.53 (11.31 – 13.75)	12.63 (12.09—13.71)	57.90 (56.81—59.99)
**SFA (g)**	5.32 (4.97 – 5.71)	5.56 ( 5.25 – 5.88)	6.03 (5.18—6.86)	5.28 (4.72 – 5.84)	5.49 (5.27 – 5.71)	28.31 (3.09—53.52)
**MUFA(g)**	3.95 (3.31—4.52)	3.82 (3.57 – 4.07)	3.94 (3.36—4.53)	3.44 (2.91 – 3.97)	3.85 (3.51 – 4.12)	30.84 (5.62 – 56.07)
**PUFA(g)**	2.43 (2.2 – 2.66)	2.47 (2.26—2.69)	2.34 (1.98—2.74)	2.46 (1.98 – 2.95)	2.44 ( 2.30 – 2.58)	29.12 (4.59 – 55.01)
**Cholesterol (g)**	63.7 (53.49—70.61)	61.68 (56.12 – 67.23)	62.56 (48.3—77.18)	67.06 (52.02 – 82.09)	62.78 (58.28 – 66.41)	218.99 (192.06 – 245.90)
**Ca (mg)**	187.43 (177.9—196.32)	196.17 (186.3 – 205.73)	193.4 (176.3 – 211.81)	209.36 (165.78 – 252.95)	193.5 (186.7 – 200.27)	642.67 (612.5 – 673.42)

T-test was used to compare gender differences in energy and nutrient intake among age groups

Abbreviations: *B* Breakfast, *D* Daily

Mean value differed significantly by sex * *P* < 0.050

Table 5 indicates the mean intake of food groups in the total population and across BQI categories. Bread (*P* = 0.040), cheese, green leafy vegetables, red vegetables, fruits, energy (*P* < 0.001 for all) and carbohydrate (*P* = 0.006) intake increased across BQI categories, however, sugar consumption was decreased across BQI score (*P* = 0.032).

**Table 5:** Mean food groups, macronutrient and energy intake in breakfast in association to BQI score and BQI categorised

	**Mean (95%CI)** **g/B**	**BQI categories**			***P* trend**
		**Low**	**Medium**	**High**	
		**(0-3points)**	**(4–6 points)**	**(7–10 points)**	
		***n* = **231	***n* = **529	***n* = **90	
		**Mean (95%CI)**	**Mean (95%CI)**	**Mean (95%CI)**	
**Bread **^a^	40.33 (38.29—42.76)	35.32 (31.76—40.17)	41.92 (38.06—44.50)	44.39 (38.76–41.47)	0.040*
**Other Grain **^b^	2.35 (2.01- 3.61)	1.45 (0.29—2.11)	2.98 (2.14—3.72)	1.57 (0.64 -2.85)	0.035*
**Cheese **^a^	15.33 (14.80 -16.74)	12.92 (10.86 -13.92)	16.55 (15.33 -16.81)	18.92 (16.86—20.57)	< 0.001*
**Egg**	7.35 (6.42—9.81)	7.98 (6.24—9.74)	7.65 (6.83—8.90)	8.62 (6.86—9.48)	0.771
**Low Fat Milk **^b^	11.86 (9.54 -13.71)	8.45 (4.31–12.59)	13.45 (10.33 -16.81)	7.45 (2.83 -12.51)	0.036*
**High Fat Milk**	0	0	0	0	-
**Butter**	1.59 (1.32 -1.79)	1.82 (1.46—2.27)	1.45 (1.33—1.81)	1.23 (1.06—1.42)	0.146
**Solid Oil**	0.32 (0.26—0.49)	0.25 (0.1—0.41)	0.39 (0.26—0.52)	0.18 (0.03—0.32)	0.073
**Liquid vegetable oil**	0.73 (.61—0.79)	0.7 (0.54—0.86)	0.73 (0.61- 0.81)	0.52 (0.30—0.74)	0.491
**Poultry** ^c^	1.18 (0.71—2.06)	1.05 (0.33—2.01)	1.04 (0.63—1.58)	0.35 (0.02—0.81)	0.005*
**Red meat**	1.39 (0.91–2.67)	1.45 (0.33—11.51)	1.26 (0.33—10.84)	1.80 (0.33 -12.36)	0.801
**Processes meat**	0.28 (0.08—0.71)	0.18 (0.03—0.50)	0.38 (0.04—0.76)	0	-
**Legume**	0.55 (0.42—0.71)	0.67(0.20—1.42)	0.55 (0.36—0.84)	0.35 (0.03—0.41)	0.189
**Nuts**	1.32 (0.8—1.89)	1.52 (0.03—2.74)	1.29 (0.73 -1.81)	1.03 (0.65—1.49)	0.643
**Red vegetable **^a^	5.34 (4.19—6.58)	0.68 (0.04 -1.42)	5.41(4.05—6.89)	11.79 (7.06—16.72)	< 0.001*
**Green leafy vegetable **^a^	4.93 (3.60—5.57)	0.87 (0.13 -1.58)	6.23 (4.98—7.71)	15.03 (10.30—19.65)	< 0.001*
**Other vegetable**	0.32 (0.01—3.45)	0	0.34 (0.06—0.68)	0	0.289
**Fruits and fruits juice **^a^	3.45 (2.09—4.19)	0.36 (0.06—0.77)	4.92 (3.86—5.22)	6.55 (3.33—7.51)	< 0.001*
**Sugar **^c^	8.41 (8.01—9.76)	9.76 (8.43 -10.75)	8.47 (7.49—8.98)	7.21 (2.37—11.41)	0.032*
**Sweet and cake**	6.94 (5.73—9.72)	9.65(6.75 -12.83)	5.87 (4.33 -7.91)	6.15 (2.63—11.01)	0.163
**Salty snack**	0.04 (0.01- 0.61)	0	0.06 (0.02—0.16)	0	-
**Black tea**	148.32 (145.11—154.50)	152.32 (141.4—157.52)	148.33 (141.11—155.13)	137.20 (124.1–144.2)	0.215
**Herbal tea**	0.27 (0.03—0.59)	0	0.42 (0.06—0.92)	0	-
**Coffee**	0.59 (0.21—0.89)	0.53 (0.06 -1.21)	0.45 (0.01—0.88)	0.87 (0.03—2.51)	0.901
**Energy **^a^**(Kcal/B)**	416.1 (411.6—419.4)	374.6 (360.06—398.14)	428.9 (415.11—441.08)	438.5 (414.6—461.20)	< 0.001*
**Carbohydrate **^a^	66.48 (64.80—69.53)	61.28 (57.06—65.03)	63.32 (65.83 – 69.93)	70.79 (66.50 -74.13)	0.006*
**Protein**	13.03 (12.31–13.86)	12.13 (10.13 -13.26)	13.50 (12.31 -14.06)	13.93 (12.33—13.89)	0.318
**Fat**	12.63 (12.09 -13.71)	12.01 (11.09 -13.01)	13.53 (12.39 – 13.66)	13.58 (12.29—14.52)	0.551

Mean and confidence interval 95% (CI 95%) are presented. ANOVA analysis was used. The Bonferroni's multiple comparison was applied for comparing means across categories of BQI

*Abbreviations: g/B* gram intake per breakfast, *Kcal/B* kilocalorie intake per breakfast

^a^ Means are significantly increased across BQI categories (*p* < 0.05)

^b^ Means are significantly different between Low and Medium BQI categories (*p* < 0.05)

^c^ Means are significantly decreased across BQI categories (*p* < 0.05)

^*^ *P* value is considered significant at < 0.05

Table 6 shows the association between food group intake in breakfast and BQI score across the time-related eating patterns. Linear regression analysis *R*^2^ adjusted = 0.004; *P*value < 0.001). First time EO was negatively associated to protein (β (CI 95%) =—0.12 (-0.004- -0.0006); *R*^2^ adjusted = 0.03; *P*value = 0.011), fat intake (β (CI 95%) =—0.09 (-0.0003—-0.00008); *R*^2^ adjusted = 0.091; *P*value = 0.023) and BQI score (β (CI 95%) =—0.14 (-0.0001- -0.00006); *R*^2^ adjusted = 0.015; *P*value < 0.001). Last time EO showed a negative association to vegetable intake (β (CI 95%) =—0.14 (-0.0005- -0.00007); *R*^2^ adjusted = 0.020; *P*value = 0.001) and BQI score (β (CI 95%) =—0.15 (-0.0003- -0.00006); *R*^2^ adjusted = 0.015; *P*value < 0.001)). length of nightly sleep associated positively to BQI score (β (CI 95%) = 0.08 (0.0002- 0.006); *R*^2^ adjusted = 0.015; *P*value = 0.004) and negatively grain intake in breakfast (β (CI 95%) = -0.06 (-0.00002- -0.000003); *R*^2^ adjusted = 0.020; *P*value = 0.032). Frequency of meals was positively associated with BQI score (β (CI 95%) = 0.21 (0.003–0.021); *R*^2^ adjusted = 0.019; *P*value < 0.001), energy (β (CI 95%) = 0.14 (0.001- 0.09); *R*^2^ adjusted = 0.024; *P*value < 0.001), carbohydrate, β (CI 95%) = 0.11 (0.0004–0.007; *R*^2^ adjusted = 0.030; *P*value = 0.012), protein β (CI 95%) = 0.07 (0.0002–0.001; *R*^2^ adjusted = 0.012; *P*value = 0.022) and fat intake (β (CI 95%) = 0.09 (0.0003–0.006); *R*^2^ adjusted = 0.0141; *P*value = 0.006). chronotype was not significantly associated with breakfast quality and food group consumption.

**Table 6:** Mean food groups, energy and macronutrient intake daily and in breakfast with fasting window, eating window, first-time EO, last-time EO, length of sleep, frequency and chronotype score

	**Mean (95%CI)**	**fasting window**		**Eating window**		**First-time eating occasion**		**Last-time eating occasion**		**Length of nightly sleep**		Frequency **meals**		Chronotype	
		β	P	β	P	β	P	β	P	β	P	β	P	β	P
**Grain group (g/B)**	57.8 (55.34—60.41)	0.01	0.921	0.001	0.990	0.005	0.912	0.02	0.602	-0.06	0.042^**a**^	- 0.01	0.718	- 0.04	0.081
**Fruits group (g/B)**	3.54 (2.90—4.88)	0.01	0.001^**b**^	-0.11	0.001^**b**^	0.005	0.897	0.01	0.793	0.009	0.081	0.019	0.587	0.01	0.382
**Vegetable group (g/B)**	10.34 (9.31–12.71)	0.12	0.010^**c**^	- 0.11	0.011^**c**^	- 0.04	0.247	-0.14	0.001^**d**^	- 0.04	0.219	- 0.006	0.972	0.005	0.875
**Dairy group(g/B)**	26.59 (24.76—28.9)	0.06	0.061	- 0.06	0.059	0.01	0.651	- 0.07	0.072	0.04	0.163	0.03	0.293	- 0.04	0.661
**Meat group(g/B)**	10.47 (9.65—11.90)	0.05	0.252	- 0.04	0.247	0.001	0.712	0.014	0.854	0.02	0.824	0.72	0.424	- 0.01	0.674
**Energy (Kcal/B)**	416.1 (411.6—419.4)	0.07	0.134	- 0.02	0.241	-0.06	0.215	- 0.03	0.346	-0.06	0.083	0.14	** < **0.001^**e**^	- 0.03	0.423
**Carbohydrate (g/B)**	66.48 (64.80—69.53)	0.03	0.446	- 0.03	0.417	-0.008	0.845	- 0.02	0.493	-0.04	0.196	0.11	0.012^**f**^	- 0.03	0.275
**Protein (g/B)**	13.03 (12.31—13.86)	0.42	0.371	0.04	0.373	-0.12	0.011^** g**^	- 0.08	0.846	-0.04	0.235	0.07	0.022^** h**^	0.03	0.427
**Fat (g/B)**	12.63 (12.09 -13.71)	0.03	0.429	0.04	0.414	-0.09	0.023^**i**^	- 0.02	0.502	-0.07	0.032^**j**^	0.09	0.006^** k**^	0.04	0.301
**BQI score**	4.54 (4.45—4.65)	0.39	** < **0.001^ l^	- 0.40	** < **0.001^** l**^	-0.14	** < **0.001^** m**^	-0.15	** < **0.001^** m**^	-0.08	0.004^**n**^	0.21	** < **0.001^**o**^	0.04	0.185

Linear regression analyses model. Adjusted: Age, gender, physical activity, educational level, occupation, smoking status, energy intake, supplement intake and BMI

Significant associations shown in bold

*R*^2^ adjusted = a 0.007; b 0.011; c 0.020; d 0.020; 0.024; f 0.030; g 0.012; h 0.012; i 0.091; j 0.020;; k 0.141; l 0.004; m 0.015; n 0.019; o 0.03

## Discussion

We investigated the association between time-related meal pattern and breakfast quality. The linear regression analysis was adjusted for the potential confounders indicated that a longer fasting window was associated with better breakfast quality. However, a wider eating window was related to lower breakfast quality. Participants with earlier first time EO had higher BQI scores and greater consumption of protein and fat in their breakfast. Greater meal frequency was also associated with higher breakfast quality and macronutrient intake at the breakfast. In addition, longer nightly sleep length was associated with better breakfast quality. The energy intake in breakfast and the ratio of breakfast energy intake to daily energy was significantly higher in men than women. Sixty two and two percent of participants had medium breakfast quality. Younger adults had better breakfast quality than older.

We found that earlier first meal and last meal consumption was associated with better breakfast quality. In line with our findings, a negative association between the time of the first meal and daily diet quality [52] was reported in pregnant women. The earlier food consumption results in better satiety and hunger control during the day [53, 54], leading to the intake of the earlier last high-caloric foods [8], and less often skipping breakfast [55]. The earlier breakfast intake may also occur without time pressure that could result in a better food quality and quantity intake in the morning. The time of food consumption could affect overall intake, and eating a large meal in the morning could reduce the overall intake throughout the day [56]. Previous studies showed that eating late may have an impact on the daily rhythms of the peripheral clock [57] and alert the daily rhythm of salivary microbiota diversity [58].

We also found that longer nightly fasting duration was associated with better breakfast quality. However, fasting duration did not show a significant association with overall diet quality in Gontijo research [52]. Shorter eating window is related to greater breakfast quality in the current study. Although the longer eating duration is a negative factor for metabolic health [59], it is associated with better overall diet quality [52]. Previous results have shown that nighttime eating [60] could increase cardiometabolic risk by disrupting circadian rhythms [61, 62]. In contrast, randomized controlled trials of Intermittent fasting and time-restricted feeding were used as weight reduction sterategy [63].

Another finding of this study was a positive association of meal frequency with a better breakfast quality and higher macronutrient intake in the morning meal. Previous studies showed a higher frequency of meals was associated with higher fruits and vegetable intake, higher overall diet quality [52, 64], and higher nutrient density [65]. Intake of smaller multiple meals is related to attenuation in insulin response and releasing gastric hormones [66] and then the positive effect on satiety. A study showed that one extra meal per day (1 extra meal/day) increased the HEI-2015 score by 3.6, although associations between snack frequency and diet quality varied depending on the definition of snacks [67]. Another study showed meal frequency but not snacks positively was associated with nutrient intake and overall diet quality [68]. Breakfast is defined as the first meal of a day broken fasting after a long period of sleep and intaked within 2–3 h after waking up. It can contain at least one food group or beverage consumed at any location [46, 69]. Generally, breakfast is consumed in the morning by most people, although it might be consumed later by shift workers and people who sleeped during the day. In some previous studies, breakfast was known as all foods and beverage consumed between 6:00—9:00 AM [70], 5:00—10:00 [71], 5:00—10:30 [45] and 5:00 – 11:00 [38, 72]. Some studies also defined breakfast according to calorie intake [16, 73]. In this study breakfast was defined based on calorie intake and time of consumption. Support for the excellent time of breakfast is limited, although it could influence the association between the number of meals and diet quality because of the lack of a standard definition of meals.

We also found that the length of nightly sleep was related to better breakfast quality. It is reported that habitual breakfast consumers had better sleep quality compared to those skipping their breakfast [74]. Shorter sleep duration was associated with lower energy intake at breakfast [75] and lower daily diet quality [76]. Sleep quality was also associated with dinner time, bedtime and breakfast frequency among Iranian [77]. Food consumption or omission of the wrong biological time results in misalignment circadian and sleep–wake up disturbance [78]. Quality and quantity of sleep may change appetite for breakfast meal in the morning [79]. In the current study, chronotype did not show any significant association with breakfast quality. Previous studies showed that evening type participants consume a unhealthier diet [31] and intend to skip breakfast [80] and have a late lunch and dinner or delay in meal timing compared with morning types [81]. Combinations of meal frequency, sleep quality, meal timing, and nightly fasting time independently and through their effects on diet quality may change satiety hormones (leptin and ghrelin), improve the peripheral circadian clock (improve metabolic regulator) and reduce oxidative damage [38].

This study is the first among Iranian adults that assessed the association between timed-related meal patterns and breakfast quality. However, some limitations of this study should be considered in the interpretations of results. This study was a cross-sectional study, and it is challenging to derive causal relationships from a cross-sectional. We used 24hDRs, a short-term dietary assessment method that provides more detailed information about amounts of food than long-term assessment method [82]. However, it has been shown that 24hDRs are related to a large within-person variation of dietary estimates. Moreover, previous studies assessing the validity of three 24-hDRs had indicated mixed results [83, 84], especially among populations with heterogeneity. All self-reported dietary assessment methods have measurement errors, but 24-hDRs are a better measure than FFQ and also, different from FFQ, allow for meal analysis [85]. Misreporting of dietary intake is a serious problem associated with self-reported dietary assessment methods [85]. Additionally, the breakfast definition is inconsistent across studies which could affect results.

## Conclusion

Longer fasting window and nightly sleep length, earlier first and last meal intake, and greater meal frequency were associated with higher breakfast quality among Iranian adults. A longitudinal study is suggested for a better understanding of the association between time related meal pattern, diet quality and health outcomes.

## Data Availability

The datasets generated and analyzed in current study are available from the corresponding author (SSb) upon request with reasonable justification. The data are not publicly available because they contain confidential information that may compromise the privacy/consent of the participants.
